# Multiple Inertial Measurement Unit Combination and Location for Center of Pressure Prediction in Gait

**DOI:** 10.3389/fbioe.2020.566474

**Published:** 2020-10-29

**Authors:** Chao-Che Wu, Yu-Jung Chen, Che-Sheng Hsu, Yu-Tang Wen, Yun-Ju Lee

**Affiliations:** Department of Industrial Engineering and Engineering Management, College of Engineering, National Tsing Hua University, Hsinchu, Taiwan

**Keywords:** inertial measurement unit, center of pressure, long short-term memory, sensor placement, gait

## Abstract

Center of pressure (COP) during a gait cycle indicates crucial information with regard to fall risk such as balance capacity. The drawbacks of conventional research instruments include inconvenient use during activities of daily living and expensive costs. The present study illustrates the promising fall-relevant information predicted by acceleration and angular velocity data from different placement sensors with machine learning techniques. This approach is inspired by the emerging machine learning technique, specifically the long short-term memory (LSTM), which is often used in time series data and aims to decrease the burden of the user while using the novel wearable technology. The Jaccard similarity coefficient, which implies the consistency of profile alignment between prediction and real situation, achieved 94% accuracy in the walking direction. Furthermore, the number of sensors used and the placement influenced the feasibility of an application. The outcome revealed that the accuracy could exceed 90% with only one sensor placed on the foot in the walking direction, and the toe would be the best location for sensor placement. To examine the performance of machine learning, the current study employed two parameters from different perspectives. One is a commonly used parameter, which represented the error, and the other investigated the similarity between the prediction and ground truth. From a similarity perspective, the parameter can be used as a metric to assess the consistency of profile alignment.

## Introduction

During a gait cycle, the center of pressure (COP) is essential information from heel strike to toe off in a walking step. COP is often considered as an indication of balance capacity, and changes in COP are widely used to differentiate physiological impairments in the fields of medicine, rehabilitation, etc., for gait analysis (Winter, 1995). For example, the COP trajectory in step-page gait was shown to be significantly different from normal gait and established a clear relationship with drop foot (Stornelli, 1990). Moreover, the velocity and displacement in specific sections of COP during gait initiation have been used to identify improvements after Tai Chi training in the elderly (Hass et al., 2004). During the stance phase, COP can be further divided into several sub-phases, and changes in the proportion of each sub-phase were used to reflect different walking speeds (Chiu et al., 2013). Hence, the parameters derived from COP, such as the velocity in different sub-phases or area ratio between medial and lateral directions, were validated, and its reliability indicates these parameters as useful features for gait analysis (Cornwall and McPoil, 2000, 2003).

Several instruments, like force plate, pressure mat, or pressure insoles, are used to measure ground reaction force (GRF) for COP trajectories during gait (Chen and Bates, 2000; Yao et al., 2010). Force plate and pressure mat consistently provide accurate GRF data, but the space of measurement is restricted since they are fixated on the ground and could only acquire a limited number of steps (Han et al., 1999). Alternatively, pressure insoles might be the most suitable among the above instruments for measuring COP in daily activity. However, being short lived, unavoidable decline in sensing capacity over time, and the high costs of pressure insoles make long-term monitoring of COP in real-life environments challenging (Shahabpoor and Pavic, 2017). On the other hand, a sensor, Inertial Measurement Units (IMUs), with an accelerometer for linear acceleration, a gyroscope for angular velocity, and a magnetometer for magnetic orientation, has become a popular instrument for gait analysis. IMU sensors were employed and attached to the body segments for GRF prediction (Charry et al., 2013; Leporace et al., 2015; Guo et al., 2017). Among these studies, the accuracy of vertical GRF predictions ranged from 3.5 to 6.8% body weight via machine learning.

Recently, machine learning emerged as the method of option for gait analysis. Leporace et al. (2015) used multilayer perceptron networks to predict tri-axial ground reaction force (GRF) based on IMU data. Choi et al. (2019a) compared the performance of the feed-forward artificial neural network (FFANN) and long short-term memory (LSTM) in the prediction of complete gait cycle COP based on single-stance COP. Of the various machine learning algorithms, LSTM is often used in time series data and showed better performance than others (Gers and Schmidhuber, 2000; Zhao et al., 2018). In addition, when using IMU data for predictions in gait, these IMU sensors were set from one to six and the locations ranged from head to foot (Leporace et al., 2015; Anwary et al., 2018; Shahabpoor and Pavic, 2018). Different locations of IMU sensors have been reported that could influence the interpretable information for gait analysis; even when five sensors were all placed on a foot, only a single IMU was used for GRF predictions (Anwary et al., 2018). A step further with GRF, COP trajectories reflect the balance, pathological state, and neural control of human gait (Winter, 1995) and could be predicted by IMU data (Leporace et al., 2015). On the other hand, the IMU sensor acted as a wearable device, which means that it can only function when worn on the human body. If multiple sensors are required for acceptable estimations of gait parameters and the placement locations interfere with daily activities, it would negatively impact the willingness to use wearable devices. However, the number and the location of IMU sensors for high-accuracy COP predictions remain unclear. The current study aims to predict COP by conducting various combinations of IMU(s) set at different locations with the LSTM models. Three locations of foot, toe, lateral, and heel, were selected. From a biomechanic perspective in gait, the foot would attach to the floor during the stance phase, and less information acquired from IMU sensors mounted on the foot. The toe IMU sensor was expected to receive information slightly after heel strike at the beginning of the stance phase. At the late stage of the stance phase, movements could be firstly observed at the heel after heel off occurs. The lateral sensor could partly cover the information of the beginning and late stages of the stance phase. An additional location at the waist level was picked to complement the small acceleration and gyroscope value during the stance phase.

## Methods

### Experimental Procedure

Five healthy participants (age: 25 ± 1.87 years, height: 1.71 ± 0.06 m; weight: 55.2 ± 5.45 kg) participated in the experiments. All participants were free from any musculoskeletal disorder and neurologic disease that could affect their performance of the experimental tasks. The project was approved by the Institutional Review Board of National Tsing Hua University, and all participants provided written informed consent before taking part in the experimental procedures. The experiment was performed in accordance with relevant guidelines and regulations.

**FIGURE 1:**
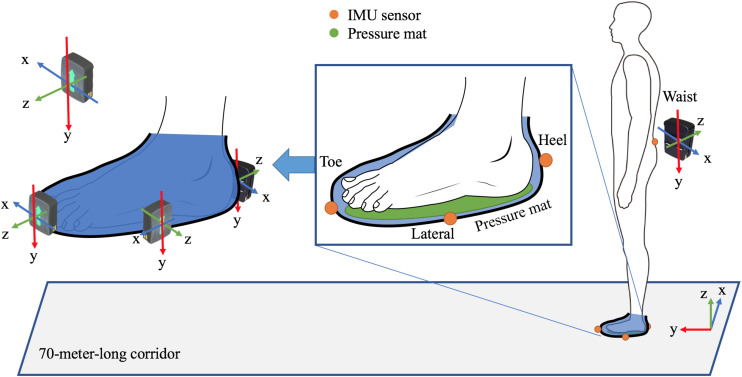
The experimental setting includes three IMU sensors attached on the left toe, lateral, and heel parts of an experimental shoe and an additional IMU sensor attached at the waist level and two pressure mat set beneath both feet.

The experimental setting is shown in Figure 1. Three IMU sensors (Delsys Inc., United States) with tri-axial accelerometer and gyroscope, which have a measuring range of ±16*g* (accelerometer) and ±2000 degrees per second (gyroscope), were attached on the left toe, lateral, and heel parts of an experimental shoe, and an additional IMU sensor was attached at the level of the waist, while an insole pressure mat (Tekscan, Inc., United States) consisting of 3.9 resistance-based sensels per square centimeter, with a sensing range from 75 to 125 psi, was set beneath both feet measuring COP with regard to ground truth. This study trimmed the mat according to the size of every participant, and the insole pressure mat was equilibrated using a step calibration according to the guidelines of the manufacturer. The participant stood on one foot for a pre-defined time and then the measurements would be normalized by the calibration result. Both IMU and pressure mat sensors were re-sampled at 148 Hz. In the experiments, the participants were instructed to walk back and forth along a 70-m-long corridor with their self-selected comfortable walking speed twice. One trip yielded 80 steps, and thus, approximately 160 steps were collected. This study also eliminated the first and last three steps in one trip. Therefore, the 74 steps of the left foot for each subject were considered to be the final data.

### Data Process

**FIGURE 2:**
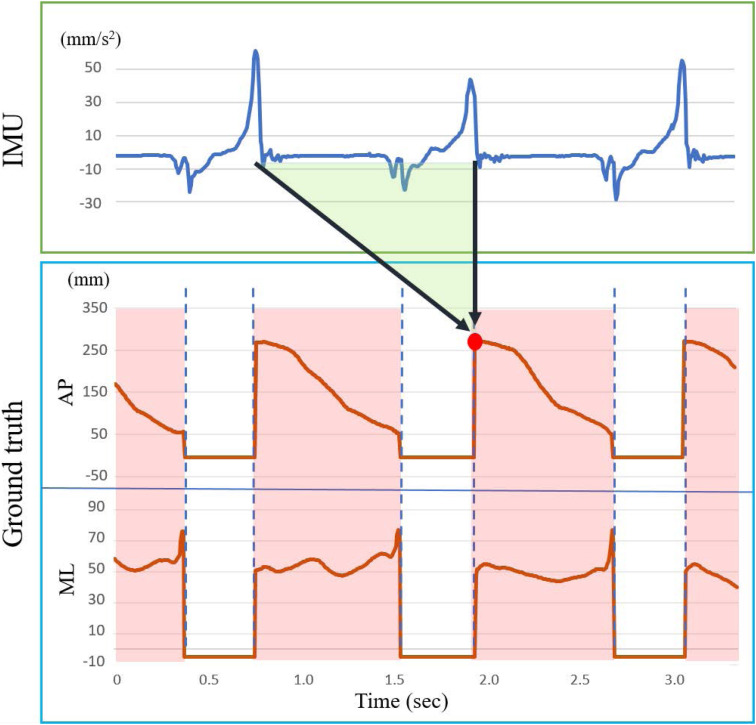
An example illustrates the COP prediction in the long short-term memory model. The green trapezoid shadow is the input feature data. A red rectangle shadow between two blue vertical dashed lines is the ground truth COP data. The red spot represents the corresponding predicted spot.

All sensors were synchronized by the first peak signals from heel IMU and insole pressure mat induced by stomping the floor at the beginning of each walking trip. COP trajectory could only be calculated in the standing phase during a gait cycle, and plantar pressure should be zero during the swing phase. Values above zero during the swing phase were considered to be noise and eliminated before inputting them to the machine learning model. The GRF data were used to extract the standing phase period measured by the pressure mat to avoid the effect of zero value received in the swing phase. A threshold of 10 N and 25 N GRF was used to detect the heel strike and the toe off as the starting and ending point of the standing phase, which has been employed for gait event determination via force platform and insole pressure (Hreljac and Marshall, 2000; Tirosh and Sparrow, 2003; Hunter et al., 2005; Catalfamo et al., 2008). The prediction of COP using IMU sensor data was a sequential problem such that temporal information needs to be taken into consideration. The IMU sensor data including previous swing phase period would be used to predict the COP trajectory of the following standing phase (Hua et al., 2019). A three-layered LSTM model was used and the ground truth COP was divided into anterior–posterior (Muro-De-La-Herran et al., 2014) and medial–lateral (ML) directional information. The way of the training LSTM model is illustrated in Figure 2. A time step period of IMU data (green shadow) prior to the predicted COP point (red spot) was taken as input data, and the stance phase COP data were extracted (red shadow) as ground truth. Subsequently, input IMU data and the predicted point were moved forward frame by frame to finish the whole COP prediction. The time step period is defined as the average of all training step lengths of the participant individually calculated from heel strike to the next heel strike. Seventy percent of around 74 steps were randomly selected to be training data, and the remaining 30% were testing data. The LSTM model is composed of three hidden layers and another dropout layer. Three hidden layers of 64/128/64 neurons were used; epoch and batch size were set to 20 and 148, respectively. The number of neurons and epoch was determined by trial and error to minimize the root mean square error values, and the batch size was set to 1 s, which can cover the whole stance phase. Loss function and optimizer of root mean square error and RMSprop were used, and the dropout layer was used to avoid over- or underfitting. The LSTM models would be conducted individually for the 1-IMU set of heel (H), lateral (L), toe (T), and waist (W); the 2-IMU set of H+L, H+T, H+W, L+T, L+W, and T+W; the 3-IMU set of H+L+T, H+L+W, H+T+W, and L+T+W; and the 4-IMU set of H+L+T+W. The 15 predicted COP trajectories were used for evaluation of the accuracy.

### Evaluation

The normalized root mean square error (NRMSE) shown in the following equation was calculated to analyze the accuracy of the COP predication. NRMSE was calculated for the AP and ML directions separately. The NRMSE equation is as follows:

$$NRMSE\left(\%\right)=\frac{\sqrt{\left(\sum_{0}^{t_{\mathrm{end}}}\left[{\left({\mathrm{COP}}_{\mathrm{meas}.}\left(t\right)-{\mathrm{COP}}_{\mathrm{pred}.}\left(t\right)\right)}^{2}\right]\right)/N}}{\max{\mathrm{COP}}_{\mathrm{meas}.}\left(t\right)-\min{\mathrm{COP}}_{\mathrm{meas}.}\left(t\right)}\times 100$$

where *N* is the sample size being extracted starting from 0 to end, which is 120 in the current dataset. The larger NRMSE value indicates the larger deviation between the ground truth and predicted COP.

**FIGURE 3:**
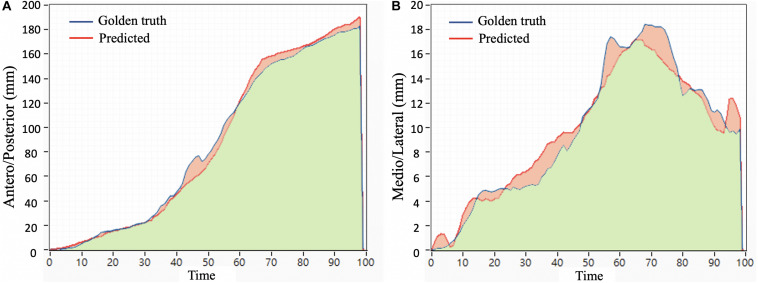
An example of the Jaccard similarity coefficient is an area under the ground truth and predicted COP in **(A)** the anteroposterior direction and **(B)** the mediolateral direction.

The similarity between the ground truth and predicted COP is determined using the Jaccard similarity coefficient (Hunter et al., 2005; Anwary et al., 2018; Shahabpoor and Pavic, 2018) and calculated for the AP and ML directions, respectively. The Jaccard similarity coefficient is defined as the size of intersection (green shadow area) divided by the size of union (green shadow area plus red shadow area). Before the calculation, original points of both predicted and ground truth COP were shifted to zero. The Jaccard equation is as follows:

$$J\left(A,B\right)=\frac{A\cap B}{A\cup B}$$

where *A* and *B* are the ground truth and predicted COP, respectively. Figure 3 illustrates the ground truth (blue) and predicted (Markowitz and Herr, 2016) COP in the AP direction from one participant for one step. The green area represents the overlap area under both COP trajectories.

## Results

**FIGURE 4:**
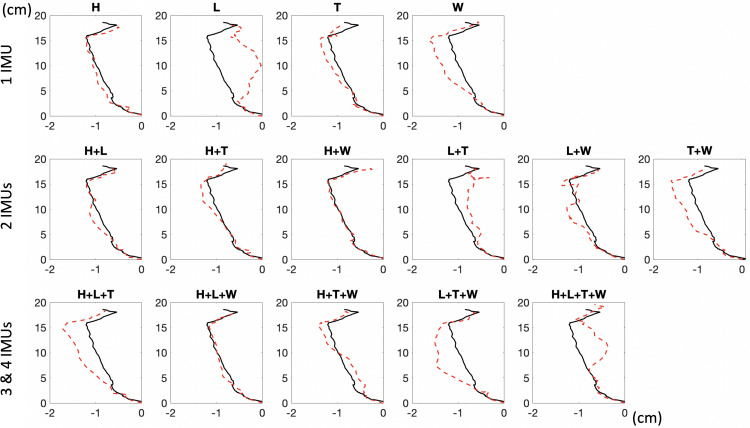
The ground truth (black lines) and predicted (red dashed lines) COP for 15 combinations of IMU sets are from one representative participant. H, heel; L, lateral; T, toe; W, waist.

Figure 4 shows a typical example of the predicted and measured COP trajectory for 15 different combinations of IMU set. Black lines are ground truth values from pressure insole sensors, and red dashed lines are predicted results from IMU data. The best prediction for the one-IMU set is the H set, that for two-IMU set is the H+W set, and that for the three- and four-IMU set is the H+L+W set based on the visualization in Figure 4. Take this representative participant as an example. The ground truth COP length for averaged steps was 17.48 cm in the AP direction and 1.48 cm in the ML direction. The predicted COP length averaged throughout all IMU combinations was 18.10 cm in the AP direction and 1.51 cm in the ML direction.

The NRMSE averaged throughout all IMU combinations was evaluated for each participant and showed that the NRMSE was remarkably smaller in the AP direction than in the ML direction. In the AP direction, the maximum mean NRMSE was 7.30 ± 1.79% observed from participant 5, and the minimum mean NRMSE was 4.29 ± 1.22% from participant 1. Meanwhile, the maximum and minimum mean NRMSE in the ML direction were observed from participant 2 (31.70 ± 3.80%) and participant 5 (15.52 ± 3.20%). Overall, the mean NRMSE was 5.88 ± 0.96% in the AP direction and 25.33 ± 2.35% in the ML direction. Alternatively, the average NRMSE over five participants for different IMU combinations was also calculated in the AP and ML directions. In the AP direction, the smallest NRMSE was 4.37 ± 1.24% from the H+T set, and the largest was 8.02 ± 1.51% from the W set. In the ML direction, the smallest NRMSE was 21.55 ± 4.35% from the L+T+W set, and the largest was 29.05 ± 8.58% from the H+L+T set.

**FIGURE 5:**
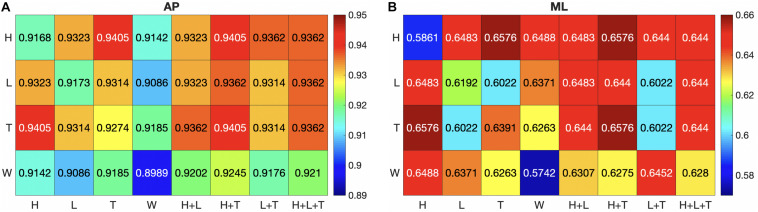
The mean Jaccard index of the ground truth and predicted COP is calculated for each combination of IMU sets in the AP **(A)** and ML **(B)** directions. H, heel; L, lateral; T, toe; W, waist.

The mean Jaccard index for each IMU combination was shown in the AP direction and the ML direction (Figure 5). For the mean Jaccard index, the lowest was observed from the W set, and the highest was from the H+T set in both directions. The highest Jaccard index was the T set when using the 1-IMU combination, the H+T set when using the 2-IMU combination, and the H+L+T set when using the 3-IMU combination in the AP direction. In the ML direction, the same 1-IMU and 2-IMU combinations were observed, but it was the L+T+W set when using 3-IMU combination. Overall, the mean Jaccard index averaged throughout all IMU combinations was 0.9269 in the AP direction and 0.6335 in the ML direction. Moreover, the highest and lowest Jaccard index was 0.9632 from the H+T set and 0.8694 from the W set in the AP direction. The highest Jaccard index was 0.7952 from the H+L+T+W set, and the lowest was 0.3891 from the H set in the ML direction.

## Discussion

The current study aimed to employ IMU data for COP prediction in gait and evaluate the estimation of IMU combinations at different locations. The estimation accuracy was much better in the AP direction (NRMSE: 6% and Jaccard index: 93%) than in the ML direction (NRMSE: 25% and Jaccard index: 63%). According to the Jaccard index, the best combination for COP prediction was observed at the H+T set while the worst one was the 1-IMU set at the waist level for both directions.

The more IMU sensors combined set was expected to provide better COP prediction, however, the best prediction came from the 2-IMU combination, and even the 1-IMU set was better than the 4-IMU set, except for the W set. A sensor at the waist level was originally treated to complement the small acceleration and gyroscope value during the stance phase, but the combination containing the waist sensor made the worse prediction. The sensor attached to the lower extremity has been reported to provide more information in gait than at the waist level (Storm et al., 2016). Furthermore, the estimation accuracy of gait spatiotemporal parameters decreases with the increase of distance from the IMU sensor to the ground when compared to the sensors on the trunk, thigh, or shank with feet (Langley, 1994; Lau and Tong, 2008; Catalfamo et al., 2010; Mannini and Sabatini, 2012; Mariani et al., 2013). On the other hand, machine learning aims to address datasets that are excessively large and complex. Although the more IMU sensors are used in data acquisition, the more information can be provided into the prediction algorithm, not all data are important for the algorithm. If too many irrelevant features are taken as inputs in the model, they would be treated as noise and would compromise the relevant information from the rest of the data. Elimination of irrelevant features would be crucial for improving the performance of machine learning (Langley, 1994). The current outcomes confirmed that more IMU sensors did not provide the higher accuracy of COP prediction; instead, less is better.

With respect to sensor placements on the foot, the IMU data of the sensor placed on the toe revealed all the best prediction combinations for the 1-, 2-, or 3-IMU set. From a kinematic point of view in gait, the whole foot is dynamic during the swing phase, and all IMU sensors receive changes in acceleration and angular velocity regardless of the location of the toe, lateral, or heel. However, during the stance phase, the heel part of the foot becomes static after the gait phase of initial contact on the ground until the gait phase of heel off. Therefore, the IMU on the heel is relatively silent during this period. Similarly, the lateral part of the foot and the IMU on the lateral side are static and silent during the midstance phase. On the contrary, the toe remains dynamic during the entire stance phase; even the heel starts to leave the ground while the metatarsophalangeal joints flexed (Miyazaki and Yamamoto, 1993; Kim et al., 2012). Hence, the movement of the toe is more abundant than other sets during gait and comprises more characteristics of individuals. The best location for placing an IMU to predict the COP trajectory would be the toe part of the foot.

Machine learning models are widely applied in predicting gait information, and the LSTM model showed better predicting results than other artificial neural network methods (Choi et al., 2019a, b; Hua et al., 2019). In the current study, a three-layered LSTM model was also built for predicting COP, and the many-to-one predicting strategy was used, which means only one COP data would be received in each prediction. The strategy was due to the measurement mechanism of IMUs (received signal only in the swing phase) and pressure mat (received signal only in the stance phase). It allowed the model to finish the prediction of each step without needing to normalize its time length to a fixed window in advance. Therefore, the LSTM model could keep the time length information successfully. For evaluation of the difference, the NRMSE of the predicted GRF and the ground truth value were calculated as an indication of accuracy performance (Shahabpoor and Pavic, 2018), while the Jaccard index can be used as a metric to assess the consistency of profile alignment (Charalambous and Bharath, 2016). The present study employed these two different parameters for appraising each combination. In COP trajectories, NRMSE represents the error between prediction and ground truth, however, the Jaccard index implies how similar the pattern of prediction and ground truth is. Both showed consistent evaluation in the AP direction, but not in the ML direction. As the pattern was similar in the AP direction, the error was small and vice versa, which was not the case in the ML direction.

Walking is essentially forward progression in the AP direction. The displacement, velocity, and acceleration in the ML direction are relatively small, consistent, and almost negligible compared to all in the AP direction (Perry and Davids, 1992; De Cock et al., 2008). Therefore, the information related to the ML direction that IMU could contribute to COP estimation was considerably fewer than the AP direction. On the other hand, the conventional way to calculate the COP trajectory from the force plate includes force and moment components in both directions (Winter et al., 1996). IMU data have been widely employed to predict GRF and show robust performance in the vertical direction (Charry et al., 2013; Guo et al., 2017; Shahabpoor and Pavic, 2018). However, for the other directions, it has been reported that the medial/lateral GRF could result in substantial variation (John et al., 2012) or overestimation using the IMU approach (Ancillao et al., 2018; Wu et al., 2020). Indeed, higher error rates of GRF prediction in the AP and ML directions were observed by using IMU data when sensors are located beneath the walking surface (Wu et al., 2020). In the current study, the greater deviation of COP prediction was also observed in the ML direction compared to the AP direction. Furthermore, the initial point was aligned to zero of the predicted and ground truth COP trajectories for Jaccard calculation. Therefore, the Jaccard index might become an index for quantifying the performance of COP prediction from the perspective of profile alignment. Together, it might explain the bad prediction performance and inconsistency between NRMSE and the Jaccard index for the ML direction.

The study has some limitations. First, only five subjects were measured in the study. However, the input matrix had a size of 38,332 (number of frames: 38,332 = 5 subjects × 74 steps × 148 frames × 70% for training dataset) × 6–24 (number of input features, which depended on the IMU combination). The input matrix size might be considered comparable to the previous study of estimating the inclination angle between the center of mass and COP (36,000 × 9), which involved 24 subjects (Choi et al., 2019a). However, the input matrix provided sufficient within-subject variability, but not between-subject variability, due to the limited number of subjects. Second, data from young, healthy subjects were only conducted in the present models. Therefore, generalization to other populations should be considered with care, and more subjects might be required for further investigation to verify the consistency of the prediction outcomes.

## Conclusion

The current study demonstrated the promising COP prediction in the AP direction by different combinations from the data of IMU sensors with LSTM models. Furthermore, the outcomes also revealed that the irrelevant features would compromise the real information from the rest of the inputs, for example, the IMU sensors at the waist level. Finally, based on the kinematics of the toes during gait, the IMU sensor on the toe could comprise more characteristics of individuals. If only one IMU could be worn to predict the COP trajectory, the toe would be the best location for placing an IMU.

## Data Availability Statement

The original contributions presented in the study are included in the article/supplementary material, further inquiries can be directed to the corresponding author.

## Ethics Statement

The studies involving human participants were reviewed and approved by National Tsing Hua University. The patients/participants provided their written informed consent to participate in this study.

## Author Contributions

C-CW and Y-JC wrote the manuscript. C-SH carried out the experiment. Y-TW and Y-JC undertook the data analysis. Y-JL supervised the project and revised the manuscript. All authors contributed to the article and approved the submitted version.

## Conflict of Interest

The authors declare that the research was conducted in the absence of any commercial or financial relationships that could be construed as a potential conflict of interest.
